# The Belgian Virtual Tumorbank: A Tool for Translational Cancer Research

**DOI:** 10.3389/fmed.2019.00120

**Published:** 2019-05-31

**Authors:** Kim Vande Loock, Eva Van der Stock, Annelies Debucquoy, Katia Emmerechts, Nancy Van Damme, Etienne Marbaix

**Affiliations:** ^1^Belgian Cancer Registry, Brussels, Belgium; ^2^Service d'Anatomie Pathologique, Université Catholique de Louvain, St-Luc University Hospital, Brussels, Belgium

**Keywords:** catalog, tumorbank, data quality, virtual, cancer

## Abstract

**Background:** Biobanks play a critical role in cancer research by providing high quality biological samples for research. However, the availability of tumor samples in single research institutions is often limited, especially for rare cancers. In order to facilitate the search for samples scattered among different Belgian institutions, a nationwide virtual tumorbank project was launched and is operational since February 2012. The Belgian Virtual Tumorbank (BVT) network encompasses the tumor biobanks from eleven Belgian university hospitals that collect and store residual human tumor samples locally and is coordinated by the Belgian Cancer Registry.

**Materials and Methods:** A web application was developed and consists of two modules. The registration module (BVTr) centralizes the tumor sample data from the local partner biobanks. The catalog module (BVTc) allows researchers to trace the tumor samples in the 11 tumor biobanks. The BVTc contains patient, medical and technical data, but excludes identifying information to ensure privacy of individuals. Automatic and manual controls guarantee high quality data on the samples requested by scientists for research purposes in oncology. A major advantage of the BVT network is that the available data can be linked to the data of the Belgian Cancer Registry for quality control purposes.

**Results:** Currently, more than 92,000 registrations are available in the catalog. Twenty-seven percent of the residual primary tumor samples originate from breast tissue, but also less frequent localisations such as head and neck (4%), male genital organs (1.7%), and urinary tract (1%) are available. In addition to the residual tumor tissue samples, also other available material can be stored and registered by the local biobanks. The most common type is corresponding normal tissue (19%).Other frequently available materials are plasma, blood, serum, DNA, and buffy coat. Even PBMCs, RNA, cytology, and urine are available in some cases.

**Discussion and Conclusion:** The BVT catalog is a valuable source of information for oncology research and the ultimate goal is to promote multidisciplinary cancer research (i.e., pathogenesis, disease prediction, prevention, diagnosis, treatment, and prognosis) for the benefit of all cancer patients.

## Introduction

Cancer registration in Belgium has evolved from a number of regional initiatives in the late nineties toward a national and centralized population based cancer registry with a firm legal basis. In 2003, the Royal Decree on the oncological care programs describing reimbursement of the multidisciplinary team meeting, was enacted (1). Later on, in 2006, the specific law on the Cancer Registry was created, making cancer registration compulsory for the oncological care programs and for the laboratories for pathological anatomy (2). The Belgian Cancer Registry (BCR) is a population-based registry that reports regularly on cancer patterns and trends in incidence and cancer survival, giving insights in the role, objectives and dataflow of the Cancer Registry (3–11). The patient's unique national social security identification number (SSIN) enables linkage with other medical and/or administrative data sources and allows the patient's vital status follow-up (12). In addition to describing cancer incidence and survival, the BCR is also involved in clinical registration projects (13, 14), in the evaluation of quality of care in oncology (15), in the registration of all tissue samples taken for early diagnosis and screening for breast, colorectal and cervical cancer (16) and in the centralization of the data on residual human tumor samples stored in local biobanks for scientific research purposes.

A biopsy or resection of tissue for diagnostic or therapeutic purpose might result in left over tissue or residual tissue. Instead of discarding this valuable material, this residual tissue can be stored at the local biobank together with associated clinical data and can be used at a later timepoint for scientific research. According to the Belgian Royal Decree on biobanks (17), every patient admitted to the hospital must be informed about the potential use of this residual tissue in scientific research. This is often mentioned in the welcome brochure of the hospital (= presumed consent). In case of explicit explanation and signing of a document, this is called an informed consent. Opposition needs to be communicated to the treating physician after which all residual tissue and associated data from the involved patient will be destroyed.

Different aspects of modern biobanking were recently highlighted by Paskal et al. (18) and the critical role of biobanking in cancer research by providing high quality biological samples for research has been shown by various papers (19–21).

In Belgium, a first biobank network was created in 2007 as a collaboration between 5 university hospitals. The network gathered pathologists and oncologists to discuss and evaluate the biobanking situation. This first consortium adopted the model of a virtual biobank and set objectives in order to extend the project to all major university hospitals in Belgium. In the course of the next years the network expanded, leading to the current network of 11 university hospitals. These hospitals all have a local biobank which stores human residual material, including tumor samples.

In March 2008, the National Cancer Plan (NCP) was launched by the former federal minister of Social Affairs and Public Health (Minister L. Onkelinx). One of the funded initiatives was the creation of a Belgian Virtual Tumorbank (BVT) in order to promote translational cancer research and the collaboration between different cancer researchers in Belgium^1^. Coordination of the Belgian Virtual Tumorbank was assigned to the Belgian Cancer Registry. A Steering Committee was setup with representatives of all biobanks for the strategic management of the Belgian Virtual Tumorbank. The criteria for recognition and conditions for the hospitals to be financed by this initiative are stated in the Royal Decree of September 20th 2009 (22).

The aim of the BVT is to facilitate the search for tumor samples scattered among different institutions by centralizing the data of residual human tumor samples in one database. A coded version of this central database is made available in an online application, which is called the BVT catalog (BVTc), and is accessible to researchers in the broad field of oncology. This application allows the researchers to perform queries based on specific search criteria to locate the samples of their interest in the different Belgian local tumorbanks. Afterwards, the researchers can contact the involved biobanks to get access to the samples. Since the BVT is fully integrated in the Belgian and European Biobank Network (BBMRI.be and BBMRI-ERIC), researchers that do not find suitable samples for their research in the BVT catalog, can be directed to the BBMRI-ERIC Directory of European biobanks^2^ and the linked Negotiator service.

This paper gives an insight in the dataflow of the Belgian Virtual Tumorbank and the different quality control steps that are performed in order to guarantee high quality of data about human tumor samples in the BVT.

## Materials and Methods

### Data Flow and Quality Control

For the setup of the BVT a custom-made online application was developed, consisting out of 2 modules; the registration module (BVTr) or central database and the catalog module (BVTc). Both applications can only be accessed by authorized users after identification and authentication by a user and access management (UAM) system to allow highly secure handling of medical data. The use of medical data in this initiative is authorized by the Data Protection Authority (23).

**Figure 1:**
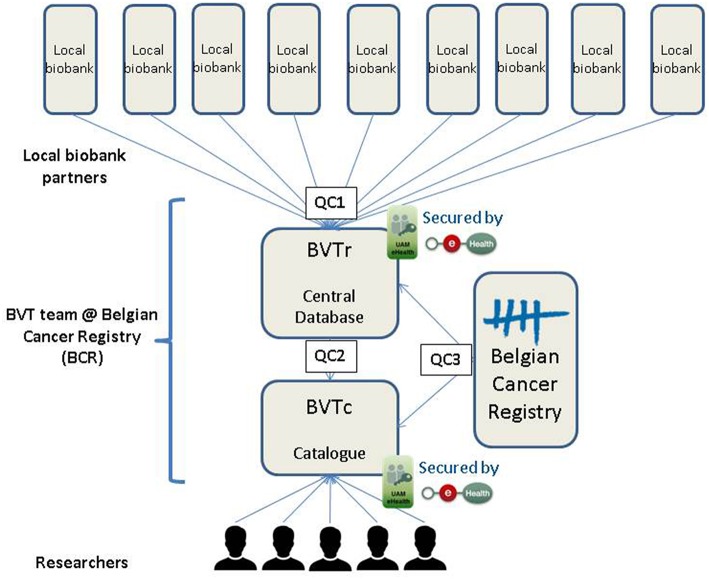
Data flow and quality control steps of the Belgian Virtual Tumorbank.

Before researchers are allowed to trace tumor samples of their interest at the local biobanks, three steps need to be performed (Figure 1). The first one is registration of the necessary data regarding the residual tumor samples stored at the local biobank in one central database. The second step involves processing of this data, including quality control, to allow publication in the coded database of the Belgian Virtual Tumorbank catalog (BVTc). In the third step, the researchers need to request access to the BVT catalog. By following a strict quality control (QC) incorrect data on biospecimens, which could crucially influence the research output, is limited to a minimum. Data quality of the BVT includes control measures at every stage of the data process guaranteeing a high quality of the data on the biospecimens requested for research purposes. Each of these steps will be elucidated in the next paragraphs, including the automatic and manual quality controls.

#### Registration of Data in the BVT (BVTr)

Local biobanks store residual tumor samples as well as relevant clinical and technical data regarding the samples. The residual tumor samples are collected under the condition of presumed consent in accordance with the Belgian law. For associated materials (e.g., plasma, serum, …), patients sign an informed consent in the hospital linked to the local biobank. If the patient opposes, the patient needs to inform the treating physician and all samples and data of the patient are removed.

A standard set of variables that needs to be completed for every tumor was defined by the BVT Steering Committee in 2010 (Table 1). The local biobanks can collect additional data (epidemiological data, molecular data, imaging data,…), but this data is not collected centrally in the catalog, since the authorization of the Data Protection Authority only allows the collection of the data mentioned in Table 1. Samples are registered via the online web application, which is restricted to authorized users only, because of the sensitive medical data that are available in the application. The registration module (BVTr) allows the local biobanks to upload registrations, query and update their own data if necessary. Local biobanks can enter one single registration or upload multiple registrations in batch. Most biobanks use their own local registration system to store relevant data concerning the stored residual tumor samples in a structured way. By exporting their data to a standardized template for batch upload (.csv-file) via an extraction algorithm, these biobanks can easily upload this extracted file in the central database of the BVT.

**Table 1:** Overview of the set of variables per database.

**Category**	**Local biobank**	**BVTr**	**BVTc**
Source		Laboratory	Laboratory
		Creation date	
		Update date	
		Reference ID	Reference ID
Patient	SSIN	SSIN	
	Gender	Gender	Gender
	Birth date	Birth date	
		Age	Age
	Patient opposition	Patient opposition	
Technical	Sample ID	Sample ID	Sample ID
	Biopsy number	Biopsy number	
	Sample date	Sample date	Sample year
	Conservation mode	Conservation mode	Conservation mode
	Conservation delay	Conservation delay	Conservation delay
	Vital state of the patient (at the time of resection)	Vital state of the patient (at the time of resection)	Vital state of the patient (at the time of resection)
	Available material	Available material	Available material
	Technical remarks	Technical remarks	
Oncological	Sample type	Sample type	Sample type
	Sample localization	Sample localization	Sample localization
	Localisation of the primary tumor in case of metastasis	Localisation of the primary tumor in case of metastasis	Localisation of the primary tumor in case of metastasis
	Laterality	Laterality	Laterality
	Morphology	Morphology	Morphology
	Behavior	Behavior	Behavior
	Degree of differentiation	Degree of differentiation	Degree of differentiation
	pTNM	pTNM	pTNM
	pTNM prefix	pTNM prefix	pTNM prefix
	Oncological remarks	Oncological remarks	
	Other		

On the moment of uploading the data an automatic quality check is performed by the application. This first data quality control step (QC1) includes format checks, content checks and a few basic cross checks. A format check implies that the format of the variable will be verified e.g., social security identification number (SSIN) should contain 11 digits. During the content checks, the application will verify whether the variables contain the predefined content. One of the cross checks performed by the application is checking whether the birth date of the patient precedes the sample date. If a variable is incorrect or if a mandatory variable is not filled in, the registration will not be accepted by the application and either appear in an error file visible for the local biobank (in case of a batch upload) or generate an error message (in case of submission of one registration in the BVTr-application). After this automatic validation, the registered data appears in the central database of the BVT.

#### Processing of Data From the Central Database (BVTr) to the Catalog (BVTc)

Once data are registered in the central database, BCR employees authorized to have access to the BVTr can search the uploaded data from all the local biobanks and perform a manual quality control of the registered data before this data becomes available in the catalog. During this second quality control step (QC2), the expert data manager at BCR will manually verify all variables using advanced crosschecks and registration rules to check for internal inconsistencies. When inaccuracies are noticed, the records are rejected. Rejected registrations need to be verified and corrected or confirmed by the local biobank and can then be uploaded again into the central database. If the quality of all data is good, records are published into the catalog. Publication of the data implies that the registrations will be coded and become available in the catalog.

#### Access to the Catalog of the BVT (BVTc)

The catalog contains technical, oncological and patient details but excludes identifying information; SSIN, technical and oncological remarks are removed, birth date is converted into age and sample date into sample year. In accordance with the central database of the BVT, access to the catalog is restricted to authorized users only. The BVT catalog is accessible to Belgian researchers working in the oncology field, that have a research project approved by an ethical committee, after authorization of the BVT steering committee as described in the authorization of the Data Protection Authority (24).

The BVT catalog allows researchers to perform queries based on specific search criteria and trace the samples of interest located at different local biobanks of the BVT network. Upon request from the researcher or local biobank, a third quality control step (QC3) can be performed on data of specific samples selected for their study. Via the unique patient identifier (SSIN), the expert data manager at BCR can crosslink the overlapping information present in the BVT database and the database of the Belgian Cancer Registry. The BCR database is restricted to data of malignant tumors with a structural delay of 2-years in incidence year to allow the programs to provide the compulsory information and perform treatment of the data at the BCR. If SSIN is not available (foreign patient) or in case of mismatch the patient can be traced by laboratory, biopsy number, and sample year.

Common variables between the BVT central database and the BCR cancer registration database are SSIN, birth date, gender, sample date, biopsy number, sample type, sample localization, laterality, morphology, behavior, differentiation grade, pT, and pTNM prefix. Post-surgical histopathological classification of the primary tumor according to TNM classification (pT), laterality, differentiation grade and pTNM prefix are non-mandatory fields in the BVT. Comparison of the overlapping information allows to distinguish recurrent tumors from primary tumors and provide additional information that might be of importance for the researcher like neoadjuvant treatment or the staging parameter that indicates the extent of the tumor after clinical diagnosis (cTNM). The database of the Belgian Cancer Registry is estimated to be more than 95% complete (25). Incompleteness of the database is more likely in case of elderly patients with very poor prognosis at diagnosis and patients with clinical diagnosis only.

## Results

### Description of the Tumor Sample Registrations in the BVT Catalog

Currently, the BVT catalog contains 92,164 registrations from 48,756 patients. There are 53,142 registrations from female patients and 39,022 from male patients. Twelve percent of the registrations (11,102) origin from metastases and 84.9% from primary tumors (78,664). The primary tumors can be divided into malignant (70%), *in situ* (1.5%), borderline (2.4%), and benign tumors (11%). For 55.6% of the registrations only residual tumor tissue is stored at the local biobank (Table 2). For some patients, paired samples of other material types are stored and registered besides tumor tissue. The most common type is corresponding normal tissue (19.4%), followed by blood (7.6%), plasma (7.4%), and serum (6.4%).

**Table 2:** Overview of available material.

Available materials^*^	**Number of registrations**
Only tumor tissue	62,263
Corresponding normal tissue	21,698
Blood	8,517
Plasma	8,303
Serum	7,115
DNA	2,595
Buffy coat	1,256
PBMC	144
RNA	71
Urine	68
Cytology	32
Total	112,062

* *multiple other materials can be indicated*.

Of all registrations, 69.3% (69,813) are stored at −80°C and 28.8% (28,980) are included in paraffin blocks. A small fraction (1.9%) of the fresh frozen samples are stored in liquid nitrogen. Conservation delay, time between excision of the residual tumor tissue and storage of the sample, is <30 min for 17% of the registrations (15,915) and more than 30 min for 25.7% of the registrations (23,691). For 57% of the registrations the conservation delay is unknown.

### Third Quality Control on Data of Breast Tumor Samples

This part focusses on the third step of the data quality control: comparison of the data available in BVT and in the BCR cancer registry database. Additional quality control checks have been performed on various tumor types like breast, kidney and esophagus. In this article, the results of the most recent QC3 analyses on data of breast tumor samples will be highlighted.

As far as the distribution sample localization is concerned, every localization is well-represented in the catalog of the BVT (Table 3). Breast tumor samples comprise more than one fourth (26%) of all primary tumor samples available. Therefore, we decided to perform a quality control study on data from primary breast tumor tissue samples, including patients from all 11 local biobanks. Taking into account the most recent complete and available database of the Belgian Cancer Registry at the moment of study set-up, it was decided to select patients with samples collected in 2014. A search using sample localization (breast C50), sample year (2014), behavior (malignant /3) and sample type (primary tumor) as criteria resulted in the retrieval of 2,358 tumor sample registrations. Only published registrations (i.e., available in the BVT catalog) were taken into account. Next, a random selection of 20 patients per local biobank resulted in a final study population of 197 patients. This means that not for all biobanks 20 patients were available.

**Table 3:** Overview of the topology groups.

Sample localization^*^	**Number of registrations**
Breast	20,458
Central Nervous System	7,646
Colorectal	6,684
Other Digestive Organs	5,741
Soft Tissue	5,702
Lung	5,251
Lymph Nodes	4,186
Kidney	3,762
Endocrine Organs	3,754
Female Genital Organs	3,219
Head and Neck	3,060
Bone Marrow and Spleen	2,468
Skin	2,032
Male Genital Organs	1,329
Other Intrathoracic Organs	1,199
Bone and Articular Cartilage	1,079
Urinary Tract	845
Unknown	249
Total	78,664

* *Calculated on primary malignant tumors*.

On the final study population, a quality control was performed for common variables between BVT and BCR registration database: patient variables (SSIN, gender, and birthdate), technical variables (sample date and biopsy number) and oncological variables (sample type, sample localization, morphology, differentiation grade, and pT).

Mean age of the study population is 62 years (Table 4). Seven of the registered breast tumor samples originated from male patients. The majority of the tumor samples (77.2%) were stored at −80°C and for 15.7% of the samples the time between excision of the sample and storage was <30 min. For 59.4% of the patients (*n* = 117) other material is stored at the local biobank in addition to residual tumor tissue, including corresponding normal tissue (87.2%), blood (15.4%), serum (12%), and plasma (12%).

**Table 4:** General description of the study population.

**Variables**	***n* (%)**	**mean ± S.D**.
Age	197 (100)	62 ± 13.50
Gender (female/male)	197 (96.4/3.6)	–
Conservation mode (Paraffin/ −80°C)	197 (22.8/77.2)	–
Conservation delay (≤30 min/ >30 min/unknown)	197 (15.7/31.5/53.3)	–
Available material (only tumor tissue/other material^*^)	197 (40.6/59.4)	–
^*^Corresponding normal tissue	102 (87.2)	–
^*^Blood	15 (15.4)	–
^*^Serum	14 (12)	–
^*^Plasma	14 (12)	–

n, number of patients; S.D., standard deviation; min, minutes.

Comparison of 13 variables available in both BCR and BVT database resulted in the retrieval of additional information for 17 patients and correction of data for 19 patients (Table 5). In total, the variables of 33 (16.8%) out of 197 registrations (patients) contained 1 (*n* = 30) or 2 (*n* = 3) errors. Patient variables (SSIN, birth date, and gender) were identical between both databases. Sample type and differentiation grade data were in concordance between BCR and BVT database. Technical variables revealed 3 typing errors, 2 in biopsy number and 1 in sample date. For the oncological variables, a distinction is made between mandatory and non-mandatory variables.

**Table 5:** Overview of the comparison of the overlapping data between BCR and BVT.

**Variables**	**Registered in BVT *n* (%)**	**Corrections needed in BVT *n* (%)**
SSIN^*^	197 (100)	0
Birth date^*^	197 (100)	0
Gender^*^	197 (100)	0
Sample date^*^	197 (100)	1 (0.5)
Biopsy number^*^	197 (100)	2 (1.0)
Sample type^*^	197 (100)	0
Sample localization^*^	197 (100)	1 (0.5)
Laterality	168 (85.3)	3 (1.8)
Morphology^*^	197 (100)	10 (5.1)
Behavior^*^	197 (100)	1 (0.5)
Differentiation grade	136 (69.0)	0
pT	166 (84.3)	1 (0.6)
**Additional information (pTNM prefix)**		**pTNM prefixes to be added in the BVT** ***n*** **(%)**
Recurrent tumors (rpTNM)		4 (2.0)
Tumor resection after neoadjuvant therapy (ypTNM)		13 (6.6)

n, number of patients;

* *mandatory variable*.

For the mandatory variables the error rate is the highest in morphology with discordances in 10 out of 197 patients. Sample localization and behavior showed each only 1 error. For one patient the tissue sample concerned a skin tumor instead of a breast tumor as was registered in the BVT database.

As far as the non-mandatory variables are concerned, the pT was incomplete for 31 out of 197 patients: for 17 the pT variable was empty, for 14 patients pTx was indicated and for 1 the completed pT value was incorrect. Comparison of the laterality between both databases revealed a mistake for 3 patients out of 168 where the variable was specified. Linkage the BVT database to BCR database resulted in retrieval of additional information to complete the BVT database. For 4 patients the stored tissue concerned a recurrent tumor, while 13 patients received neoadjuvant therapy prior to resection.

## Discussion and Conclusion

The national virtual tumorbank has been set up in order to facilitate the search for samples scattered among different Belgian institutions. To achieve this an online BVT application was developed consisting out of two modules; the central database (BVTr) and the catalog (BVTc). The central database (BVTr) allows centralization of patient, technical and oncological data of human residual samples stored locally in a harmonized and standardized way while the catalog (BVTc) enables researchers to localize the samples required for their oncology research. Implementation of automatic and manual data quality control steps guarantees a high quality of associated data from residual tumor samples.

Establishment of a data standard enables biobanks to integrate within a network and allows communication not only between biobanks but also between initiatives and most importantly with researchers (26). Within BVT the standard set of variables regarding the residual tumor samples stored at the local biobanks include patient, oncological and technical information. In the BVT catalog identifying data is excluded to ensure that core information related to the patient and the sample can be found by the researcher while maintaining confidentiality of the patient. Overview of the data available in the BVT catalog shows coverage of a broad range of sample types with samples originating from primary malignant tumors, *in situ*, borderline and benign tumors as well as samples from metastases. Most of the registered samples are stored at −80°C while a significant smaller fraction is stored as paraffin-embedded blocks. This can be explained by the fact that at some local biobanks the paraffin-embedded blocks are stored at the anatomopathological department and therefore not registered in the BVT database. In addition, not all of the local biobanks have the facility to create paraffin-embedded blocks on site at the biobank. In this last years, the frozen and paraffin-embedded tumor samples and corresponding normal tumor tissue collections are more and more complemented by matched samples of blood, serum, plasma, and other body materials.

The value of successful linkage of data in biobanks and cancer registries has been elaborated in various studies (27, 28). The Belgian Cancer Registry is a population-based cancer registry collecting information on all cancer cases diagnosed in Belgium provided by the oncological care programs in all hospitals and services for pathological anatomy. Validity and quality of the data are ensured by an extended set of automated and manual validation procedures based on the IARC guidelines (29). Comparison of the overlapping information between the BVT and the BCR database revealed a good quality of sample data. Moreover, this comparison resulted in the retrieval of additional information on recurrent breast tumors and neoadjuvant therapy prior to resection of the breast tumor which might be important information for researchers. These results indicate the relevance of a joint evaluation of biobank and cancer registry information to guarantee a high quality of associated data from biospecimens used in translational cancer research. Harmonized storage of clinical and other associated data in combination with the good quality of the data might facilitate further linkage to additional information in the future. Linkage of samples to relevant clinical and (molecular) pathological information enables researchers to further understand tumor development, response to treatment and clinical outcomes (19, 20, 30).

One biobank cannot always provide sufficient numbers of samples and therefore the number of data sharing initiatives increases, enabling researchers to find suitable number of available samples and associated data (18, 19, 31–34). The developed online BVT application is a dedicated mechanism for researchers to localize their residual tumor samples of interest and associated data stored at the 11 local biobanks. The system takes into account the balance between the burden of data entry for the biobank manager providing adequate information for the researcher to find and localize their sample of interest while maintaining the confidentiality of the patient. An advantage is that the quality of the sample data of all 11 biobanks is verified in a uniform way and that the standards are extended to the application using automatic and manual quality checks. This combination of automatic and manual quality checks guarantees a high quality of the data.

## Data Availability

All datasets generated for this study are included in the manuscript and/or the supplementary files.

## Author Contributions

EV is mostly involved in data management of the BVT project and helpdesk for the application. AD supervises the coordination of the BVT project and is consulted as a biobank expert. NV was consulted and contributed as an expert of the Belgian Cancer Registry. EM is the president of the BVT steering committee and consulted as a pathology expert. KE supervises coordination of the BVT project. KV is involved in BVT project management and took the lead in writing the manuscript with input, comments, and critical feedback provided by all other authors mentioned. All authors contributed to the final version of the manuscript.

### Conflict of Interest Statement

The authors declare that the research was conducted in the absence of any commercial or financial relationships that could be construed as a potential conflict of interest.

## Footnotes

^1^www.virtualtumourbank.be

^2^https://directory.bbmri-eric.eu/
